# A Multicenter, Open-Label, Single-Arm Phase I Trial of Dual-Wield Parenchymal Transection: A New Technique of Liver Resection Using the Cavitron Ultrasonic Surgical Aspirator and Water-Jet Scalpel Simultaneously (HiSCO-14 Trial)

**DOI:** 10.7759/cureus.49028

**Published:** 2023-11-18

**Authors:** Ko Oshita, Shintaro Kuroda, Tsuyoshi Kobayashi, Gaku Aoki, Hiroaki Mashima, Takashi Onoe, Norifumi Shigemoto, Taizo Hirata, Hirotaka Tashiro, Hideki Ohdan

**Affiliations:** 1 Department of Gastroenterological and Transplant Surgery, Graduate School of Biomedical and Health Sciences, Hiroshima University, Hiroshima, JPN; 2 Department of Biostatistics, Clinical Research Center, Hiroshima University, Hiroshima, JPN; 3 Department of Surgery, Hiroshima City Hiroshima Citizens Hospital, Hiroshima, JPN; 4 Department of Surgery, Kure Medical Center and Chugoku Cancer Center, National Hospital Organization, Kure, JPN; 5 Translational Research Center, Hiroshima University, Hiroshima, JPN

**Keywords:** open liver resection, water-jet scalpel, cavitron ultrasonic surgical aspirator, anatomical hepatectomy, liver tumor

## Abstract

Purpose: This study evaluated the safety and feasibility of a technique of liver resection named dual-wield parenchymal transection technique (DWT), using cavitron ultrasonic surgical aspirator (CUSA) and water-jet scalpel simultaneously.

Methods: This multicenter, prospective, open-label, and single-arm phase I trial included patients aged 20 years or older with hepatic tumors indicated for surgical resection and scheduled for open radical resection. This study was conducted at two institutions affiliated with the Hiroshima Surgical Study Group of Clinical Oncology (HiSCO). The primary endpoint was the proportion of massive intraoperative blood loss (≥ 1000 mL). The secondary endpoints were the amount of blood loss, operative time, parenchymal transection speed, postoperative complications, and mortality. The safety endpoints were device failure and adverse events associated with devices.

Results: From June 2022 to May 2023, 20 patients were enrolled; one was excluded and 19 were included in the full analysis set (FAS). In the FAS, segmentectomy was performed in nine cases, sectionectomy in four cases, and hemihepatectomy in six cases. Radical resection was achieved in all patients. Intraoperative blood loss greater than 1000 mL was observed in five patients (26.3%). The median amount of blood loss was 545 mL (range, 180-4413), and blood transfusions were performed on two patients (10.5%). The median operative time was 346 minutes (range, 238-543) and the median parenchymal transection speed was 1.2 cm^2^/minute (range, 0.5-5.1). Postoperative complications of Clavien-Dindo classification ≥ Grade 3 occurred in four patients (21.1%). No mortalities occurred in this study. In the safety analysis, there were no device failures or adverse events associated with devices.

Conclusions: This study demonstrated the safety and feasibility of DWT for liver resection. The efficacy of the DWT will be evaluated in future clinical trials.

## Introduction

Liver resection is an established curative treatment method for benign, and primary and secondary malignant hepatic tumors [1]. In the past decades, the outcomes of liver resection have significantly improved, and advances in patient selection, perioperative management, and surgical techniques and devices have contributed to short- and long-term outcomes. Prolonged operative time and increased intraoperative blood loss are independent risk factors for postoperative complications including post-hepatectomy liver failure (PHLF) and bile leakage [2]. The occurrence of postoperative complications is associated with poor prognosis [3]. Therefore, safer and more efficient hepatectomy method has been a great concern for liver surgeons.

The method of liver resection can be divided into the two main processes: parenchymal transection and hemostatic coagulation. Parenchymal transection method has progressed with the development of various devices and techniques since the introduction of the crush clamp technique in the 1970s [4]. However, there is no established consensus on the most effective parenchymal transection method.

The cavitron ultrasonic surgical aspirator (CUSA) is a widely used device in parenchymal transection worldwide and is preferred to avoid vascular injury owing to the advantage of isolating intrahepatic vasculatures [5]. The two-surgeon technique (TST), in which the primary surgeon operates the CUSA to transect the parenchyma and dissect the vascular structures and a second surgeon directs hemostasis on the liver resection surface using the saline-linked electric cautery (SLC), was first described in 2005 [6]. The TST contributes to reducing the intraoperative blood loss, operative time, and postoperative bile leakage by allowing two surgeons to participate simultaneously in parenchymal transection and hemostasis [7,8].

A water-jet scalpel (WJS) breaks apart the liver tissue using a high-pressure water jet and selectively isolates small vascular and biliary structures, potentially decreasing blood loss and preventing thermal damage to the surrounding tissues. It has been reported that WJS is superior to CUSA in terms of the blood loss and transection speed because of the accurate identification of portal and hepatic vein structures deep within, and the removal of the parenchyma [9-11].

We hypothesized that the simultaneous use of the CUSA and WJS would allow for safer and more efficient parenchymal transection, taking advantage of the strength of each device. Therefore, we developed “dual-wield parenchymal transection (DWT)”, a new technique in which a third surgeon performs parenchymal resection using WJS in addition to conventional TST using CUSA and SLC by two surgeons. This multicenter prospective trial aimed to investigate the safety and feasibility of DWT for liver resection for patients with hepatic tumors.

## Materials and methods

Trial design

This multicenter, open-label, single-arm, prospective phase I clinical trial was conducted from June 2022 to May 2023 to evaluate the safety and feasibility of DWT for liver resection. This study was conducted at Hiroshima University Hospital (Hiroshima, Japan) and Kure Medical Center (Kure, Japan), affiliated with the Hiroshima Surgical Study Group of Clinical Oncology (HiSCO), and the study protocol was approved by the institutional review board of Hiroshima University, Japan (approval number: CRB6180006). This trial was registered with the Japan Registry of Clinical Trials (jRCTs062220031) and was conducted in accordance with the 1975 Declaration of Helsinki. All patients provided written informed consent before enrolment in the study.

Patients

Patients eligible for the study were required to meet all the following criteria: (i) Patients who were scheduled for anatomical open hepatectomy for liver tumor (indications for open hepatectomy were large tumors, multiple tumors (three or more), or tumors in close proximity to major vessels that were expected to be difficult to resect laparoscopically), (ii) Eastern Cooperative Oncology Group (ECOG) performance status of 0-1, (iii) Age ≥ 20 years at the time of informed consent, (iv) No extrahepatic metastasis from primary malignancy, and (v) No severe disturbances of blood biochemistry (white blood cell count ≥ 2000/mm^3^, hemoglobin ≥ 8.0 g/dL, platelet count ≥ 5.0×10^4^/mm^3^, total-bilirubin ≤ 3.0 mg/dL, prothrombin activity% ≥ 50%, and creatinine ≤ 2.0 mg/dL), and major organ function including the liver, lung, kidney, and bone marrow. The exclusion criteria were as follows: (i) patients who required biliary duct reconstruction, (ii) patients who required revascularization, and (iii) patients who required simultaneous surgery for organs other than gallbladder. The study data were collected and managed using the Research Electronic Data Capture (REDCap) tool hosted at Hiroshima University Hospital [12].

Surgical procedure and postoperative follow-up

The primary surgeons operating the CUSA were senior surgeons who had each performed more than 50 hepatectomies. In addition, at least one board-certified expert surgeon in the hepatobiliary-pancreatic field in Japan who had performed more than 200 hepatectomies was included in the surgical team to ensure surgical quality [13]. 

Following laparotomy, the liver was sufficiently mobilized for each surgical procedure in a standard fashion. Intraoperative ultrasonography was used to confirm the extent of the disease and to routinely plan the parenchymal transection plane. In the case of anatomical segmentectomy, intraoperative fluorescence imaging was carried out using indocyanine green injection to identify the boundaries of the segments.

**Figure 1:**
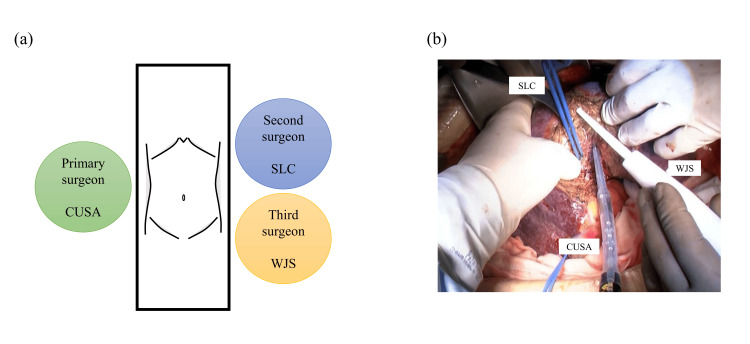
(a) Setting up the surgeon and devices for hepatectomy using DWT method; (b) Intraoperative photograph during parenchymal transection DWT, dual-wield parenchymal transection; CUSA, cavitron ultrasonic surgical aspirator; SLC, saline-linked electric cautery; WJS, water-jet scalpel

During the parenchymal transection, the primary surgeon dissected the liver parenchyma from the right side of the patient using the CUSA (CUSA® Excel Plus, Integra LifeSciences, Princeton, New Jersey, United States). The secondary surgeon performed hemostasis using bipolar soft-coagulation forceps (VIO® 300 D; ERBE Elektromedizin GmbH, Tübingen, Germany) from the superior left side of the patient. The third surgeon stood on the inferior side of the second surgeon, operated the WJS (ERBEJET®2; ERBE Elektromedizin GmbH), and dissected the parenchyma simultaneously with the primary surgeon under good visual field conditions (Figure 1 and Video 1).

**Video 1:** Parenchymal transection using the dual-wield parenchymal transection method.

Vessels smaller than 2 mm were dissected after coagulation using the bipolar coagulation forceps or dissected using the ultrasonically activated coagulating shears (Thunderbeat®; Olympus Corporation, Shinjuku City, Tokyo, Japan). Vessels larger than 2 mm were dissected after ligation with 4-0 silk. All parenchymal transections were performed under low central venous pressure (0-5 cm H_2_O) and with intermittent Pringle maneuver (15 minutes of occlusion alternated with five minutes of reperfusion). An intraoperative prophylactic drainage tube was routinely placed at the end of each procedure. The postoperative parameters of hepatic damage and recovery, including serum total bilirubin (T-Bil), aspartate aminotransferase (AST), and alanine aminotransferase (ALT) were measured on postoperative days 1, 3, 5, 7, and 30.

Endpoints

The primary endpoint was defined as the proportion of intraoperative blood loss greater than 1000 mL. The amount of blood loss was estimated by measuring the suction volume after subtracting the rinse fluids and weighing the fluid contained in the gauze used in the surgical manipulation. The secondary endpoints were the amount of blood loss, operative time, transection speed, length of postoperative hospital stay, postoperative complications, and mortality. Postoperative complications and mortality were defined as all morbidities and deaths that occurred within 30 days of surgery. The severity of postoperative complications was stratified according to the Clavien-Dindo classification [14]. Bile leakage and post-hepatectomy liver failure (PHLF) were defined and stratified for severity according to the International Study Group of Liver Surgery (ISGLS) definitions [15,16]. Parenchymal transection time was defined as the duration between the beginning and end of the parenchymal transection, including the time of the Pringle maneuver. The estimated area of the transection plane was measured using the SYNAPSE VINCENT® (Fujifilm Holdings Corporation, Minato City, Tokyo, Japan), and the transection speed (cm^2^/min) was calculated by dividing the estimated area of the transection plane by the transection time. The safety endpoint was defined as device failure and adverse events associated with the devices.

Statistical analysis

No formal power calculations were performed in this phase I study. The primary and secondary endpoints were analyzed in a full analysis set (FAS) that met all eligibility criteria, did not violate any exclusion criteria, and underwent protocol procedures. Safety was analyzed using a safety analysis set (SAS), which included all patients who underwent the protocol. All statistical analyses were performed using SAS® version 9.4 (Released 2013; SAS Institute Inc., Cary, North Carolina, United States) and JMP® version 16.0 (SAS Institute Inc.). Continuous variables are expressed as median and range. Wilcoxon signed-rank tests were used to analyze the changes in continuous data. The statistical significance was set at p-value < 0.05.

## Results

Baseline characteristics

**Figure 2:**
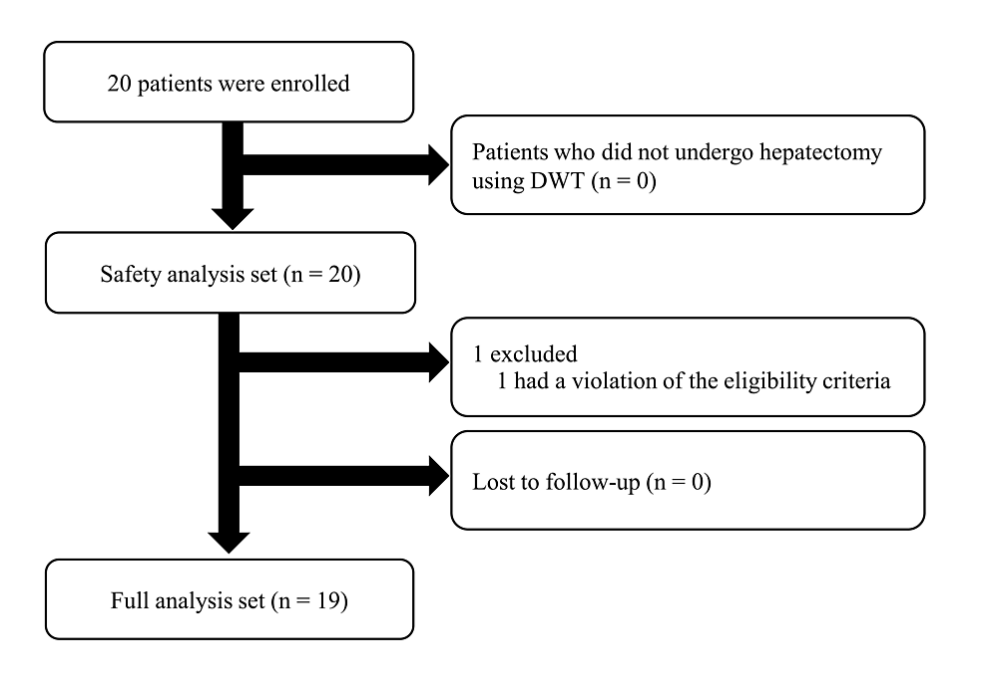
Study flow chart DWT, dual-wield parenchymal transection.

Between June 2022 and May 2023, 20 patients who underwent open hepatectomy using DWT were enrolled in this study. Twenty patients were included in the SAS. One patient was excluded because of a violation of the eligibility criteria; consequently, 19 patients were subjected to FAS analysis (Figure 2). The background characteristics of patients in the FAS are shown in Table 1. The median age of the patients was 71 years (range, 35-82); 13 patients were male (68.4%) and six patients were female (31.6%). The median body mass index was 22.8 kg/m^2^ (range, 18.9-32.2). Thirteen patients had primary liver cancer (68.4%), five patients had secondary malignant liver tumors (26.3%), and one had a hemangioma (5.3%). The median number of tumors was one (range, 1-4) and the median maximum tumor size was 40 mm (range, 10-160). Segmentectomy was performed in nine cases (47.4%), sectionectomy was in four cases (21.1%), and hemihepatectomy in six cases (31.6%).

**Table 1:** Baseline characteristics of the full analysis set. BMI, body mass index; ECOG, Eastern Cooperative Oncology Group; PS, performance status; HBV, hepatitis B virus; HCV, hepatitis C virus; T-Bil, total bilirubin, ALB; albumin; PT, prothrombin time; ICGR15, indocyanine green retention rate at 15 minutes; AST, asparate aminotransferase; ALT, alanine aminotransferase; PLT, platelet count; HCC, hepatocellular carcinoma; ICC, intrahepatic cholangiocarcinoma; CCC, combined hepatocellular and cholangiocarcinoma Variables expressed as n unless marked otherwise

Variables	n = 19
Demographic data	
Age (years), median (range)	71 (35-82)
Sex (Male/Female)	13/6
BMI (kg/m^2^), median (range)	22.8 (18.9-32.2)
ECOG-PS (0/1)	18/1
Etiology (HBV/HCV/Non-B and non-C)	2/2/15
Maximum tumor size (mm), median (range)	40 (10-160)
Tumor number, median (range)	1 (1-4)
T-Bil (mg/dL), median (range)	0.6 (0.4-1.2)
ALB (g/dL), median (range)	4.0 (2.7-4.6)
PT% (%), median (range)	92 (69-115)
ICGR15 (%), median (range)	11.0 (2.9-43.1)
AST (IU/L), median (range)	23 (13-37)
ALT (IU/L), median (range)	18 (8-45)
PLT (×10^4^/mm^3^), median (range)	16.1 (9.8-32.0)
Creatinine (mg/dL), median (range)	0.78 (0.44-1.77)
Child-Pugh classification (A/B)	18/1
Liver damage (A/B/C)	14/5/0
Diagnosis	
Malignancy (HCC/ICC/CCC/Liver metastasis)	8/4/1/5
Benign	1
Unilober/Bilober	15/4
Initial hepatectomy/repeat hepatectomy	14/5
Operative Procedures	
Segmentectomy, n (%)	9 (47.4%)
S3	1
S5	1
S6	1
S7	3
S8	3
Sectionectomy, n (%)	4 (21.2%)
Lateral sectionectomy	1
Medial sectionectomy	1
Anterior sectionectomy	2
Hemihepatectomy, n (%)	6 (31.6%)
Right hepatectomy	3
Extended left hepatectomy	1
Left hepatectomy	1
Central bisegmentectomy	1

Primary endpoint and blood loss

The postoperative findings are shown in Table 2. Five patients were observed to have an intraoperative blood loss greater than 1000 mL (26.3%), and the median amount of intraoperative blood loss was 545 mL (range, 180-4413) and two patients (10.5%) underwent intraoperative blood transfusions.

**Table 2:** Perioperative findings Variables expressed as median (range) and n (%)

Variables	n = 19
Operative findings	
Operative time (min)	346 (238-543)
Blood loss (mL)	545 (180-4413)
Blood loss ≥ 1000 mL	5 (26.3%)
Intraoperative transfusion	2 (10.5%)
Duration of Pringle maneuver (min)	69 (30-226)
Transection plane (cm^2^)	120 (30-218)
Transection time (min)	89 (13-308)
Transection speed (cm^2^/min)	1.2 (0.5-5.1)
Device failure	0 (0.0%)
Surgical margin (mm)	3 (1-12)
Radical resection	19 (100%)
Postoperative process	
Re-operation	1 (5.3%)
Mortality	0 (0.0%)
Hospital stays (day)	11 (9-116)

Secondary and safety endpoints

The median operative time was 346 minutes (range, 238-543). Median transection time and transection speed were 89 minutes (range, 13-308), and 1.2 cm^2^/minute (range, 0.5-5.1), respectively. The postoperative complication details are summarized in Table 3. Eleven of the 19 patients (57.9%) experienced any postoperative complication. Eleven complications of Clavien-Dindo grade 2 or lower occurred, and all were successfully treated with noninvasive therapy. Four postoperative complications of Grade 3 or higher occurred. One patient developed pleural effusion that improved completely with percutaneous drainage. Two patients developed bile leakage and recovered fully after percutaneous drainage and antibiotic administration without re-operation. One patient underwent re-operation for postoperative bleeding; however, the site of bleeding was an adhesion-dissected surface near the pancreas unrelated to the parenchymal transection. Of the five patients who developed PHLF, three patients had International Study Group for Liver Surgery (ISGLS) Grade A and two had Grade B. Both patients who developed bile leakage were classified as ISGLS Grade B. The median postoperative hospital stay was 11 days (range, 9-116) Radical resection was achieved in all patients without mortality. Additionally, no device failure or device-related adverse events were observed in the SAS population.

**Table 3:** Details of morbidity Variables expressed as n

Variables	n = 19
The number of patients with any morbidities	11 (57.9%)
Clavien-Dindo classification	
Grade 1	2
Anorexia	1
Anemia	1
Grade 2	11
Acute cholangitis	1
Superficial surgical site infection	1
Edema of lower limbs	1
Ascites	4
Post hepatectomy liver failure	2
Anorexia	2
Grade 3a	2
Pleural effusion	1
Bile leakage	1
Grade 3b	1
Postoperative bleeding	1
Grade 4a	1
Bile leakage	1
Grade 4b	0
Grade 5	0

Changes in liver function

**Figure 3:**
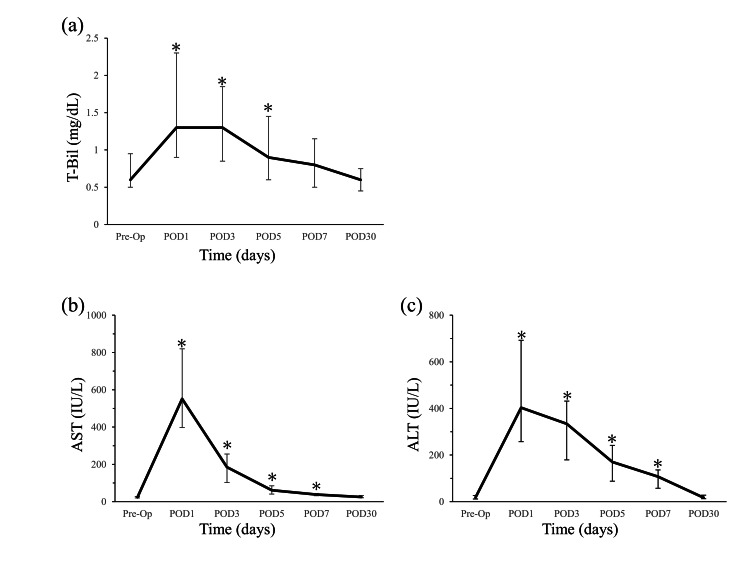
Dynamics of liver function before and after surgery: changes in (a) T-Bil, (b) AST, (c) ALT Variables expressed as the median and interquartile range; Liver function on postoperative days 1, 3, 5, 7, and 30 was compared with the preoperative data using Wilcoxon signed-rank tests. (*p < 0.05). T-Bil, total bilirubin; AST, aspartate aminotransferase; ALT, alanine aminotransferase; Pre-Op, pre-operation; POD, postoperative day

Changes in the liver function status in the FAS are shown in Figure 3. The median peak level of T-Bil, AST, and ALT were 1.3 mg/dL (0.4-3.2), 551 IU/L (range, 91-1743), and 403 IU/L (range, 42-1597), respectively; these factors were highest on postoperative day 1. Liver function on postoperative days 1, 3, 5, 7, and 30 was compared with the preoperative data using Wilcoxon signed-rank tests. There was no significant difference in the level of T-Bil on postoperative day 7 compared with the preoperative data (P = 0.08), and the levels of T-Bil (P = 0.65), AST (P = 0.49), and ALT (P = 0.87) did not differ significantly between preoperative data and postoperative day 30.

## Discussion

The present study demonstrated the safety and feasibility of DWT using the CUSA and WJS simultaneously as parenchymal transection devices in anatomical open hepatectomy. There have been no reports of techniques using CUSA and WJS simultaneously, and the main objective of this study was to evaluate the safety and feasibility of DWT, a novel surgical technique.

To perform safer and more efficient liver resection, several devices with different characteristics have been described for parenchymal transection, including crush-clamp, ultrasonic dissector, water-jet, saline-linked dissector or dissecting sealer [17]. Aloia et al. reported that TST using a combination of CUSA and SLC decreased the duration of inflow occlusion, decreased the blood loss, and shortened the operative time [6].

TST has also been used in living donor liver transplantation, and significantly reduced blood loss and donor complications in living liver resection, with no difference in early graft function and postoperative recipient survival compared to the conventional method [8]. However, anatomical liver resection is a highly invasive procedure with a high incidence of postoperative complications and mortality; additionally, the further development of surgical techniques and devices continues to improve the treatment outcomes.

The expected advantage of DWT over TST is that the use of different devices, CUSA and WJS, allows for the selection of a suitable device depending on the diameter and fragility of the vessels in isolation. WJS is characterized by a high degree of tissue selectivity during parenchymal transection and no thermal damage that causes biliary stricture [11]. The decision to increase the use of WJS during dissection of fragile smaller vessels and near the porta hepatis may reduce the intraoperative blood loss. The simultaneous use of multiple devices may also reduce the time required for the surgeon to exchange the devices. In addition, the simultaneous use of two parenchymal transection devices by two surgeons may shorten the parenchymal transection time in anatomical liver resections with large dissection areas. Parenchymal transection time using CUSA is generally slower than that using other methods, and shortening the operation time is often difficult [18,19]. Finally, it is also important that the three surgeons actively participate in the parenchymal resection. Palavecino et al. described that one of the advantages of the TST is the active participation of two surgeons during parenchymal resection [7]. Although the third surgeon who operates the WJS has a slightly caudal view of the surgical field in the DWT, surgical control can be maintained, and efficient parenchymal transection can be achieved by keeping a good surgical field of view and close communication between the three surgeons.

The primary endpoint was defined as the proportion of patients with massive blood loss greater than 1000 mL that could affect blood transfusion or postoperative complications to assess safety, rather than the total intraoperative blood loss. In this study, the proportion of patients with intraoperative blood loss greater than 1000 mL was 26.3%. In a prospective trial that evaluated the efficacy of energy devices or staplers in hepatectomy, the proportion of massive bleeding greater than 1000 mL was reported to be 15-55% [20,21]. In addition, previous studies on anatomical hepatectomy have reported a median total blood loss ranging from 435 mL to 715 mL and a transfusion rate of 17.4% [22-24]. Although this study included patients with large tumors with a median diameter of 40 mm and up to 160 mm, the blood loss and transfusion frequency were comparable to those of previously reported hepatectomies. The safety of DWT in terms of blood loss is considered acceptable, and the effectiveness of DWT in reducing blood loss may be demonstrated by comparison with other methods in a matched patient group.

There were no device failures or intraoperative adverse events associated with the devices. In addition, radical hepatectomy was completed in all patients without mortality. The changes in liver function were similar to those observed during conventional hepatectomy. Among the postoperative complications, bile leakage is a common complication after hepatectomy and is often influenced by the selection of techniques or devices for parenchymal transection. The incidence of postoperative bile leakage in this study was 10.5%. The rate of bile leakage after hepatectomy without biliary reconstruction was reported to be 3.6%-15.6% [25-27]. Anatomical hepatectomy, large tumor, or male gender have been shown as an independent risk factor for bile leakage [3,27,28]. Anatomical liver resection cases were eligible for this study, and our cohort included a high proportion of men (68.4%) and patients with large tumors up to 160 mm. Therefore, our results were considered comparable to those of previous studies. Although one patient required re-operation due to postoperative bleeding, the bleeding was on the site of the detached adhesion and was not related to parenchymal transection. Based on the results of this study, the safety of the DWT was considered acceptable.

The median parenchymal transection speed in this study was 1.2 cm^2^/minute, with a maximum of 5.1 cm^2^/minute. Although Lesurtel et al. showed the mean transection speed of CUSA and WJS were 2.3 cm^2^/minute and 2.4 cm^2^/minute, respectively, the estimated transection area was measured using a sponge and sheet of paper, which was different from our method using volume-analyzing software [29]. Since the transection speed differs depending on the method of measurement of the estimated transection area and time, evaluation under the same criteria is necessary to accurately assess the efficacy. The median transection speed of WJS in LDLT donor hepatectomy at our institution measured by the same criteria as this study were 0.66 cm^2^/min, with a maximum of 1.56 cm^2^/min [11]. A shorter transection time suggests the proper identification of the vascular structure and efficient parenchymal transection, resulting in lesser blood loss and a shorter total operative time. Although the transection speed of the DWT was better than that of the WJS in LDLT, this result did not indicate a definite benefit of the DWT because of the different subject populations. Future prospective studies comparing with other transection methods are necessary to demonstrate the efficacy of DWT in terms of transection speed.

This study has several limitations. First, the sample size was small and there was no comparison with other parenchymal resection methods. This is because the primary endpoints were defined as safety and feasibility, and further prospective comparative studies are needed to demonstrate efficacy. Second, this study enrolled patients who were not indicated for laparoscopic surgery and our cohort may have had a selection bias. Third, cost analysis was not performed. The DWT requires additional equipment for the WJS, in addition to the CUSA and SLC. However, the total cost of medical care, including intraoperative costs such as operative time or transfusions associated with blood loss, and postoperative costs such as surgical ward costs and medications, is the most important factor. The total cost can be reduced by decreasing transection time and postoperative complications. Therefore, a detailed analysis of total cost-effectiveness is needed in future studies. Fourth, the learning curve for the DWT was not considered. The learning curve is steeper in DWT than in conventional TST owing to the necessity of cooperation of the three surgeons in the management of surgical procedures, and a training period is required by participating surgeons for DWT to be effective. Fifth, the long-term oncological outcomes were not evaluated in patients with malignancies.

## Conclusions

This study demonstrated the safety and feasibility of the DWT for open anatomical hepatectomy. To the best of our knowledge, this is the first report of a surgical technique in which CUSA and WJS were used simultaneously for parenchymal transection, and DWT was considered to be effective for the parenchymal transection time and blood loss in some cases. To show the efficacy of DWT, a future prospective study focusing on total blood loss and transection speed is needed.
